# Performance and characteristics of an IR localizing system for radiation therapy

**DOI:** 10.1120/jacmp.v7i2.2190

**Published:** 2006-05-25

**Authors:** Yulia Lyatskaya, Hsiao‐Ming Lu, Lee Chin

**Affiliations:** ^1^ Department of Radiation Oncology Brigham and Women's Hospital Boston; ^2^ Department of Radiation Oncology Massachusetts General Hospital Boston Massachusetts 02115 U.S.A.

**Keywords:** posture control, respiratory control, IR system

## Abstract

We report the development of a new system for interactive patient posture, position, and respiratory control during radiation therapy treatment. The system consists of an infrared (IR) camera, retroreflective markers, and dedicated software that makes it practical to use in the clinic. The system is designed to be used with multiple retroreflective markers to monitor not only the position, but also the posture of the patient in real time. Specific features of the system include the following: (1) The system reports an absolute misalignment at several points on a patient and also provides feedback on any necessary adjustments in terms of site‐specific setup parameters, such as focus‐to‐surface distance (PIN), superior and inferior alignment, and chest‐wall angle. (2) The system is based on a set of predefined templates containing the number and position of control points and feedback parameters developed for different treatment sites. (3) A noninvasive IR‐based “virtual portal vision” procedure projects organ contours in the beam's‐eye‐view (BEV) based on the IR marker locations obtained in real time and compares them with digitally reconstructed radiographs (DRRs) from CT simulation. Assuming good correlation between external markers and internal anatomy, the system offers the possibility of mimicking a verification procedure without taking port‐films, which can potentially reduce the setup time. In this paper, we concentrate on the system properties and performance while initial applications on a number of clinical sites are ongoing. Accuracy and precision of this system are evaluated in the context of breast/chest treatments using rigid phantoms. The system has an intrinsic uncertainty of ±1mm. When two systems in different rooms (CT and treatment rooms) are used for correlating positional information, the uncertainty is less than 2 mm.

PACS number: 87.56.Da

## I. INTRODUCTION

In radiation therapy, high‐precision treatment is only possible with a high level of patient position control.^(1)^ With new advancements in treatment modalities and the increasing use of intensity‐modulated radiotherapy, the accuracy of patient positioning for the treatment becomes even more important. Lasers and field lights are routinely used for localizing an isocenter and positioning patients in the field, and portal imaging (electronic or radiographic) is used for more accurate setup verification.^(2)^ However, these techniques are limited to 2D measurements and do not easily allow for a perfect setup due to the fact that the human body is nonrigid. Patient immobilization devices such as alpha‐cradles and Vac Lock (Med‐Tec Corporation) bags improve the accuracy of patient posture control, but these devices lack quantitative feedback. In addition, large uncertainties for some treatment sites (e.g., lung and breast) remain problematic,^(3)^ especially when target volumes and/or critical organs are in motion due to breathing.^(3)^

Optical tracking has been applied to improve patient setup for radiation treatment in a number of systems (see, e.g., the recent review in Ref. 4). These systems use infrared (IR) cameras to track a number of IR‐reflecting spherical markers, affixed either to the patient's skin surface^(^ ^5^ ^–^ ^9^ ^)^ or to a rigid template,^(^ ^10^ ^–^ ^14^ ^)^ and then use these data to automatically align the patient in the desired position. Highly accurate systems have been developed first for intracranial radiotherapy^(^ ^10^ ^,^ ^11^ ^)^ and radiosurgery^(12)^ and later extended to extracranial radiotherapy applications.^(^ ^13^ ^–^ ^17^ ^)^ The main advantage of these systems is a high accuracy of isocenter correlation. These systems work extremely well for the sites with minimal organ motion, such as central nervous system sites. However, when organ motion is significant, a variation of an IR system is needed for more accurate patient setup verification. A combination of an optical system with either ultrasound^(^ ^13^ ^–^ ^16^ ^)^ or a dual X‐ray system^(^ ^6^ ^,^ ^17^ ^)^ has been developed and applied to treatment of extracranial lesions. These extra modalities are introduced, since current systems based on IR technology are mainly used for isocenter correlation only, and they do not provide sufficient information for the posture control of the patient. It was shown in the studies by Baroni et al.^(^ ^7^ ^–^ ^9^ ^)^ for breast cancer patients that the accuracy of patient repositioning is different for anatomical reference marks close to the laser marks and those farther from the laser verification points, reflecting the need for registration of the body posture in addition to isocenter verification.

In this paper, we describe a variation of an optical system designed to simultaneously monitor the accuracy of the patient's position with respect to the isocenter and the patient's body configuration. For this purpose, we differentiate between body posture and body position: posture refers to the exact configuration of the patient with respect to the table, and position relates to translational shifts from the isocenter. We optically monitor the multiple markers on the patient and utilize the ability to use the location of different markers for different purposes. Thus, the majority of the markers are used to monitor body configuration, and a single marker placed on the reference point tattooed on the skin is used to calculate required shifts relative to the isocenter. We have also integrated into our system a respiratory control function, which allows us to monitor the patient's breathing pattern in 3D space. It will be a subject of future study to evaluate whether 3D breathing control can allow for better correlation between external marker motion and internal target motion, which has been a major problem for the current gating technologies.^(^ ^18^ ^–^ ^23^ ^)^ This report focuses on the performance of the system using rigid phantom studies, demonstrating the limit of the system accuracy expected when reproducing the patient body configuration from simulation to treatment.

## II. MATERIALS AND METHODS

The main component of our system is an IR camera set that can detect 3D positions of multiple IR‐reflective markers placed on the surface of the patient's skin.

### A. IR tracking system

The basic system consists of an IR camera surrounded by a ring of IR emitters and a marker that reflects light at an angle of 180°. When the reflected IR signal is detected by an IR camera, the position of the marker can be calculated in a 2D plane. When two cameras are used, 3D coordinates of the marker can be obtained (as described in Ref. 4). We use the Passive Polaris optical tracking system (NDI model P4 position sensor) to detect 3D coordinates of several IR retroreflective markers simultaneously. This optical system has two cameras that can recognize up to 50 markers in space at a distance of 2 m to 2.5 m from the cameras with the data collection frequency of $10s^{-1}$ to $20s^{-1}$.

To express the coordinates of the markers obtained from the camera in the room coordinate system, a coordinate transformation has to be performed similar to that described elsewhere.^(24)^

Below, we will only operate within the room coordinate system, assuming that the coordinate transformation from the IR camera system to the room coordinate system has already been performed.

### B. Retroreflective markers

The markers are manufactured from thin (0.5 mm) polystyrene shells covered with a highly reflective 3M Scotchlite tape (8850 Silver Pressure Sensitive adhesive film). We use hemispherical markers 7 mm in diameter, which are mounted on a flat, thin base and are affixed to the patient's skin by adhesive film. Since the markers are hemispherical in shape, the center of the marker as detected by the optical system is located on the surface of the patient's skin and can be compared with coordinates of the same point obtained by means of other techniques (e.g., portal imager or CT if the marker is applied on the BB (a lead sphere of 1 mm diameter affixed to the patient's skin). The markers are hollow and small: hollow to eliminate the dose buildup on the patient's skin and small to avoid blocking one marker by another in the camera's viewing plane and to achieve better accuracy of marker positioning on the tattoos.

### C. System application: Simulation to treatment

**Figure 1:**
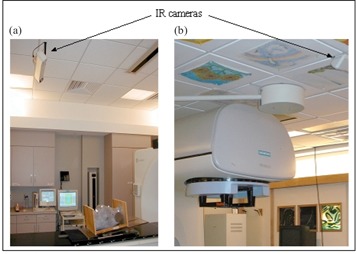
The IR system setup in (a) the CT simulator and (b) the treatment room. The breast phantom used to evaluate the accuracy of the system is also shown in (a).

At the simulation (CT or conventional), we place the markers on the patient, and the IR positioning system records the coordinates of those markers. To reproduce the patient's body configuration at treatment, an IR system identical to the one in the CT simulator is installed in the treatment room (Figs. 1(a) and (b)). During patient setup at the treatments, we place the markers at the same points on the skin (identified by using tattoos, invisible ink marks, or templates of natural skin marks). With the IR camera system, we can track the markers and compare their coordinates with the recorded values at the CT simulator so that the patient's body configuration can be accurately reproduced.

To make the setup procedure efficient, we describe the patient's body configuration by body posture and body position. The table shift (body position) can be calculated by comparing the coordinates of a single reference marker in the treatment room to the coordinates of the same marker at the time of CT simulation. Using this single marker as a reference, we can evaluate the posture of the patient based on the relative position of all other markers with respect to the selected one. To ensure fast and efficient patient setup adjustment, we transform the coordinates of the reference markers to relevant clinical parameters, for example, sternum angle (the chest‐wall angle in the superior‐inferior direction), leveling (e.g., the right‐to‐left rotation of the superior part of the chest‐wall of the patient), focus‐to‐surface distance (FSD or PIN), and table shift. Once patient posture is acceptable, the table shift is calculated and applied, and treatment can be started.

The procedure described above serves not only as a setup adjustment tool, but also potentially as a setup verification method. In current practice, port‐films or electronic portal images are used typically for verifying patient setup, one field at a time. However, these cannot be obtained until the therapists leave the treatment room, which results in a slow and noninteractive process. With a “virtual portal vision” application of our system described in Section D.6 below, we not only can instantaneously project the marker's position in BEV for patients who had CT simulation, but can also apply the same transformation to the patient's organ contours and project those in BEV (based on the position of the reference markers). We can then compare these organ projections with where they are supposed to be for a perfect setup under the assumption that the internal organ's position is correlated with the external markers. This allows a comprehensive evaluation of the patient setup for all the fields before taking any port‐film and instantaneous feedback directing the user to make any necessary adjustments on the patient. The procedure is performed in real time and is almost instantaneous, therefore potentially reducing setup time.

In order for our system to be effective, we have designed a sequenced procedure that aids the therapists as part of their treatment setup routine. This procedure is preprogrammed and automated for different treatment sites and settings, and it relies heavily on templates and database systems across the network. We also incorporate voice commands and voice instructions to make the software easier to use and operate. The software is written in Visual Basic and is Windows‐based.

### D. System performance

To verify this system's performance, we conducted several tests focusing on the accuracy of marker identification and reproducibility of the phantom setup. The performances of the camera and the camera calibration procedure are described elsewhere.^(25)^ The accuracy of the system performance is determined by the following factors associated with the main components of the system: IR camera, markers, and the phantom to which the markers are affixed (we use a rigid phantom for our tests to avoid errors introduced by the nonrigidity of the human body). We therefore can describe the accuracy of the system through three main components: (1) intrinsic accuracy of the camera set, that is, the highest possible accuracy with no error introduced by any intentional movements of the markers or the phantom (or patient); (2) accuracy of the marker repositioning on the phantom; and (3) accuracy of the phantom repositioning or transfer from one room to another. Since our system is particularly suitable for treatments that need respiratory control, we first tested it for application to chest/breast sites. To have the test geometry as close to the real breast treatment situation as possible, we used an anthropomorphic female chest phantom for all the tests. Below we describe several tests performed to estimate the accuracy of the system.

#### D.1 Intrinsic accuracy of the system for a rigid phantom

We monitored a rigid breast phantom (Fig. 1(a)) and analyzed the data for seven markers positioned with the purpose of covering different points on the phantom as shown in Figs. 2(a) and 2(b). One marker was positioned on the surface of the phantom approximately at the center of the chest area (C), three markers were positioned inferiorly (IL, IR, and IC), and three other markers were positioned superiorly (SL, SR, and SC). We were interested in this marker arrangement for the purpose of monitoring breast cancer patients. The first test was designed to verify that all markers were correctly identified (this can be a problem for the cases of a very steep chest angle). With the current position of the camera set in our treatment facility, we were able to successfully monitor all the markers within a wide range of table positions and gantry angles. With the gantry rotated to 90°, the IR‐reflective marker initially positioned at the isocenter can be tracked for all possible table positions, which is in agreement with Polaris camera specifications of an active volume of the camera being 1 m in diameter. When the gantry angle is set to 0°, the gantry inevitably blocks some of the markers. Since we position the camera to allow tracking of the isocentric marker, all markers inferior to the isocenter are also tracked. The markers superior to the isocenter cannot be seen by the camera when the table is moved vertically toward the gantry.

**Figure 2(a):**
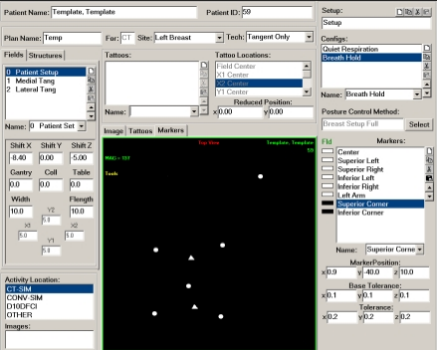
A template for marker locations and field geometry for breast cancer treatment. The central panel shows marker locations for breast treatment with field points (triangles) and posture points (circles). At the top, the patient's name and ID, plan name, treatment site, and technique are entered. Field geometry parameters are shown on the left and marker names and locations on the right.

**Figure 2(b):**
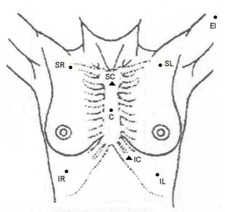
A schematic of marker locations for breast treatment. Two field points are shown as triangles: SC (superior corner) and IC (inferior corner). Six posture points are shown as circles: C (center), SR (superior right), SL (superior left), IR (inferior right), IL (inferior left), and EL (elbow).

The second test was performed to evaluate the accuracy of marker localization. We monitored this still breast phantom with the seven marker arrangement described above and collected the values of three coordinates, *x*, *y*, and *z*, for each marker for 60 s. The average values $\bar{x},\;\bar{y},\;\bar{z}$ for a set of *N* values and standard deviations from the average for each coordinate, $\sigma_{x},\;\sigma_{y}$, and $\sigma_{x}$, were then calculated, as well as the maximum deviation from the average. No intentional movements were performed during that time. Therefore, any changes in recorded coordinates can be associated only with random fluctuations and the accuracy of the IR camera coordinate calculation, that is, the intrinsic accuracy of the system.

#### D.2 Marker repositioning test

In order to estimate errors introduced by repositioning the markers on the patient's skin, we performed the following test. On an inclined immobilized surface, a mark was drawn in the same way that patients are marked in the treatment room. This is mainly done in two ways. First, some points are permanently tattooed. These points are identified before treatment, and a cross is drawn to emphasize the location of the tattoo. Second, the points that are not permanently tattooed but used for the purposes of monitoring the patient during treatment are marked with invisible blue ink. This ink is visible only under blue light and stays invisible under normal conditions. When invisible ink is used, a circle around the base of the marker is drawn at first time the marker is applied; this circle is later used for marker repositioning.

The first test involved repositioning markers on the circle. On this spot, five markers were positioned sequentially several times, 198 times in total for five markers. Every time the marker was positioned on the spot, the *x*, *y*, and *z* coordinates of the marker were recorded. Since we did not find any noticeable difference between different markers, all the data for five markers were analyzed as one set, for which standard deviations $\sigma_{x,y,z}$ from the average position in three directions were calculated.

The second test was similar to the first one except that a cross instead of a circle was used as a marker position reference. In this test, we used three markers sequentially with a total of 10 repositioning sessions.

#### D.3 Overall system performance: Transfer of phantom to another room

To evaluate the overall system performance, we conducted a test that included all the steps going from the CT simulator to the treatment room. The main component of uncertainty in this process was associated with repositioning the phantom, either in the CT simulator or the treatment room. We performed the test by monitoring seven markers affixed to the phantom in the CT simulator and using these markers to reposition the phantom based on the saved coordinates of the markers. The first test was performed in the CT simulator by repeatedly setting up the phantom guided by the IR system (to avoid error associated with laser alignment and IR camera calibration in different rooms), and the second test involved moving the phantom from the CT simulator to the treatment room. As a criterion for evaluating the quality of the setup, we compare the laser marks on the phantom for the test to those recorded initially at the CT simulator. The test was performed on an anthropomorphic breast phantom to represent realistic geometry.

At the CT simulator, we affixed several markers to the phantom, positioned the phantom in a standard way for CT scan (as shown in Fig. 1(a)), marked the intersections of the lasers on the phantom (two lateral marks and one AP mark), and recorded coordinates of the IR markers in CT room geometry with the IR cameras. A coordinate transformation was then applied to the coordinate set to obtain the markers’ coordinates for the treatment room. The phantom was then transported to the treatment room and positioned with the guidance of an identical IR system installed there (Fig. 1(b)). At first, the room lasers were turned off, and the setup was guided only by monitoring the markers on the phantom. When the treatment room lasers were turned on, we compared the marks of the lasers from the CT simulator to those in the treatment room. If the discrepancy between these marks was less then 2 mm in either direction, we concluded that the IR system performed satisfactorily.

#### D.4 Accuracy for IR‐guided setup

**Figure 3:**
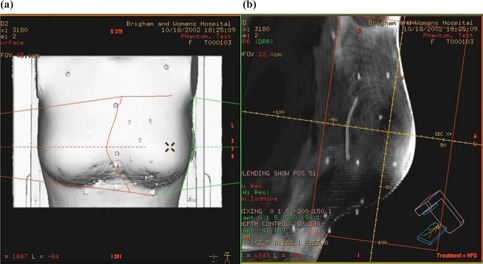
(a). The breast phantom with 10 lead BBs affixed to its surface; (b) the DRR of a medial field with BBs in beam's eye view.

The tests described in the previous sections focus on the intrinsic accuracy of an IR system alone. When this system is used in practice, the accuracy of its calibration with the lasers and the accuracy of laser alignment with the imaging plane can contribute to the total accuracy of the procedure. In this section, we describe a test to characterize an expected cumulative accuracy of the phantom setup guided by the IR system. The breast phantom previously described (Fig. 1(a)) was scanned at the CT simulator with 10 lead BBs affixed to its surface around the left breast as shown in Fig. 3(a). Two opposing fields were simulated to mimic the left breast treatment. The digitally reconstructed radiographs (DRRs) (Fig. 3b) were printed and the coordinates of BBs on DRRs were recorded. When the phantom was brought to the treatment room, it was set up, guided by the IR system. Two port‐films were taken for the medial and lateral fields. The coordinates of 10 BBs were recorded for port‐films and compared with DRRs to evaluate the accuracy of the setup.

#### D.5 Monitoring individual markers

The main focus of our new system is on monitoring and analyzing multiple markers individually rather than analyzing the ensemble of multiple markers as a whole. To test the feasibility of this application, we conducted a test on a dynamic phantom (CIRS, Norfolk, VA), which consists of a solid body with a movable insert. Four markers were attached to the solid body of a phantom, and one marker was attached to an insert moving in a predetermined wave‐form controlled by an electronic controller. The amplitude of motion was 2 cm in the cephalad‐caudad direction, and the period was 5 s. The objective of the test was to identify a single marker motion while using other markers for a setup control.

#### D.6 Virtual portal vision

We have developed an algorithm for projecting line contours of organ volumes into BEV based on the IR marker locations and comparing the projection with the reference BEV (the DRRs obtained from the CT simulation). The algorithm is based on modeling a patient as a rigid body as described in the Appendix. For example, in the thorax region this means that the patient's lung shape is assumed to be the same at the time of the treatment as it was during CT simulation, and that the lung orientation in space is correlated with the patient's chest surface orientation. When these assumptions are used, the live BEV projections of the organs are accurate only in the case of a good setup. For an incorrect setup, the BEV projections will show a mismatch of the organs indicating a potential setup problem.

With the above assumptions, we have developed a protocol that consists of three main steps:

1. At the CT simulation, (a) the optimal placement for the reflective markers is chosen and marked on the patient's skin, (b) reflective markers are placed on the skin, and (c) coordinates of these markers are recorded by the IR system and stored in the database.
2. Organ contours are obtained from CT images and imported into our software.
3. At the treatment, (a) marked points on the patient's skin are identified, (b) reflective markers are affixed to these points, (c) coordinates of these markers are recorded by the IR system, and (d) a coordinate transformation is calculated and applied to organ contours.

To verify the algorithm, we performed the following procedure. We scanned the breast phantom in the CT simulator and drew the contours representing the left lung and the tumor cavity approximately where these structures would normally be for a real patient. We also defined a field geometry that would be appropriate for the left breast treatment with the particular structures outlined. Both contours and fields were then exported to the IR system software and used to project these contours in BEV. To reconstruct the BEV projections, we assumed a rigid relationship between contours and IR reflective marker orientation in space. The following tests were performed. We affixed several markers to the phantom and monitored these markers in the CT simulator for 30 s. These recorded coordinates were then treated as the “standard.” When the phantom was moved to the treatment room, it was set up in the treatment position and the affixed markers were recorded. The coordinates of these markers were compared to the “standard” set, and displacement of the whole set of markers was calculated as a lateral shift and rotational displacement in three planes. The rotation matrixes were then calculated and applied to the CT organ contours as described in the Appendix. After this initial correction to the contour orientation, the BEV projections of these contours were calculated and both “standard” and real‐time projections displayed on the computer screen. The same process was performed for the phantom with intentional misalignment. In the case of a good setup, these projections look similar to each other; in the case of an incorrect setup, the projections should correspond to the misalignment of the phantom. This process is different from other image verification procedures that rely on either portal images,^(2)^ ultrasound images,^(13)^ or dual X‐ray verification.^(17)^ In our system, the procedure of image verification is based solely on the images (contours) obtained during CT simulation and on the IR marker location comparison at CT and treatment. This procedure is based on the assumption of a good correlation between external markers with internal anatomy; therefore, it may suffer accuracy limitations. However, it is a simple and noninvasive process that can be used as an initial setup verification procedure followed by filming the patient. It may be used on a continuous basis throughout the treatment to verify that initial setup has not changed or as a daily verification method while weekly port‐films are taken.

#### D.7 Respiratory control functions

The capabilities of this new system go beyond setup verification. With the ability to monitor points in real time, the system can be used for applications where the patient's posture changes during treatment, for example, due to respiration. This system can be used for either breath‐hold applications or gated treatments. To test system performance for respiratory control functions, we performed tests of the system on volunteers. Five markers (C, SR, SL, IR, and IL) were affixed to a person's skin in the pattern shown in Fig. 2(b). These markers were monitored with the IR camera for 20 s, and the AP displacement of the markers was plotted against time.

## III. RESULTS

Measurements to evaluate the accuracy of our system were carried out according to the tests discussed above. The results are summarized below.

### 1. Intrinsic accuracy of the system for a still phantom

The maximum deviation from the average for all markers was calculated to be 0.08 mm, and the standard deviation $\sigma_{x'y,z}$ values were as follows: $\sigma_{x}=0.01\;\text{mm},\;\sigma_{y}=0.06\;\text{mm}$, and $\sigma_{z}=0.06\;\text{mm}$ ($\sigma_{x'y,z}$ were averaged over seven markers since no appreciable difference was observed for different markers). These numbers reveal a very high intrinsic accuracy of the system, since for monitoring human patients, movements within 1 mm are considered very satisfactory in treatment setups.

### 2. Marker repositioning test

**Figure 4:**
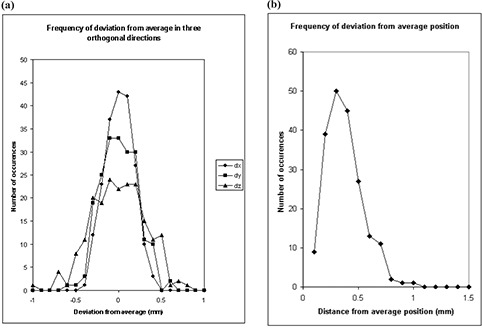
Frequency of deviation from average for 198 tests of marker repositioning on a circle: (a) deviation in three orthogonal directions, *x*, *y*, and *z*; (b) cumulative deviation from the average position.

For the marker repositioning on a circle, standard deviations were obtained as follows: $\sigma_{x}=0.3\;\text{mm},\;\sigma_{y}=0.3\;\text{mm}$, and $\sigma_{z}=0.4\;\text{mm}$, with maximum deviation from average being 0.9 mm in all three directions. Figures 4(a) and 4(b) show the frequency of deviation from the average for 198 marker repositioning sessions. Figure 4(a) shows the results for individual coordinates, *x*, *y*, and *z*, and Fig. 4(b) shows the distribution of the cumulative deviation from the average position, $\sqrt{x^{2}+y^{2}+z^{2}}$. As seen from Fig. 4(b), the most probable deviation from the average position is 0.4 mm, with the maximum observed deviation of 1.1 mm. This accuracy of marker repositioning is mainly achieved by having the circle drawn around the base of the marker. For the marker repositioning on a cross, standard deviations are $\sigma_{x}=0.5\;\text{mm},\;\sigma_{y}=1\;\text{mm}$, and $\sigma_{z}=0.4\;\text{mm}$, with maximum deviations from average being 0.7 mm, 1 mm, and 1 mm in the *x*, *y*, and *z* directions, respectively.

The above results show that the marker repositioning accuracy is worse than the intrinsic accuracy of our system by almost an order of magnitude. However, even with the highest maximum deviation from average of 1.1 mm, marker repositioning can be considered acceptable for patient setup purposes if patient monitoring tolerance is expected to be within 1 mm to 2 mm. If a higher accuracy is desirable, drawing a circle around the marker base is preferable in comparison to having a cross as a marker position reference, and a more accurate marker repositioning procedure can be designed.

### 3. Transfer of phantom to another room

Table 1a shows the discrepancies for each individual mark for the phantom repositioning tests done in the CT simulation room, and Table 1b shows same discrepancies for phantom transfer from the CT simulation room to the treatment room. The discrepancy between the marks in either direction was no more then 2 mm, indicating that the IR system can be used as a reliable setup tool.

**Table 1:** 

a. Distance from new laser mark at the CT simulator to the initial CT laser mark							
		AP (mm)		Left (mm)		Right (mm)	
Test	Location	*x*	*y*	*y*	*z*	*y*	*z*
1	CTsim	1.0	0.0	1.0	0.0	1.0	0.0
2	CTsim	0.0	1.0	−1.0	0.0	1.0	0.0
3	CTsim	1.0	−1.0	−1.0	0.5	−1.0	0.5
4	CTsim	0.0	0.0	−1.0	−0.5	0.0	−1.0
5	CTsim	0.5	0.5	0.0	−1.0	0.0	−1.0
Average		0.5	0.1	−0.4	−0.2	0.2	−0.3
b. Distance from new laser mark at the treatment room to the initial CT laser mark							
		AP (mm)		Left (mm)		Right (mm)	
Test	Location	*x*	*y*	*y*	*z*	*y*	*z*
1	Treat, room	0.5	0.5	0.0	−2.0	0.0	−2.0
2	Treat, room	0.0	0.0	−0.5	−2.0	0.5	−2.0
3	Treat, room	0.5	0.0	−1.0	−2.0	0.5	−2.0
4	Treat, room	0.0	0.0	0.0	−2.0	0.0	−2.0
Average		0.3	0.1	−0.4	−2.0	0.3	−2.0

Comparison of these tests shows that the discrepancy for phantom repositioning that comes from the IR positioning system is of the order of 0.5 mm, while a larger discrepancy (up to 2 mm) may be observed when going from room to room due to imperfections of laser alignment in each room. Since laser alignment tolerance is set to 2 mm, a discrepancy of 2 mm in phantom repositioning is not surprising.

### 4. Accuracy of the IR‐guided setup

The results for the tests described in Section D.4 are summarized in Table 2. As can be seen, the discrepancy between the BB coordinates are no more than 1 mm for the *x* coordinate in the BEV and no more than 4 mm for the *y* coordinate averaging to 0.02 mm and 0.23 mm for the *x*and *y* coordinates, respectively. The BBs closer to the isocenter show less discrepancy than those farther from the isocenter. The larger discrepancy in the *y* direction is attributed to the fact that the BBs are located farther away from the isocenter in the longitudinal beam direction.

**Table 2:** Comparison of the location of BBs on the DRR films and portal‐films taken in the treatment room

	Medial Field					
	Port‐film	SimDRR	DRR‐Sim	Port‐Film	SimDRR	DRR‐Sim
BB #	*x* (cm)	*x* (cm)	*x* (cm)	*y* (cm)	*y* (cm)	*y* (cm)
1	−3.3	−3.3	0.0	4.6	4.4	0.2
2	−0.8	−0.7	−0.1	5.0	4.8	0.1
3	−0.9	−0.9	0.0	1.2	0.9	0.3
4	1.0	1.0	0.0	−2.8	−3.1	0.4
5	−2.3	−2.3	0.0	−3.1	−3.4	0.3
6	−1.4	−1.4	0.0	−6.5	−6.8	0.3
7	−1.8	−1.8	0.0	−7.4	−7.8	0.4
8	−4.6	−4.5	0.0	−8.2	−8.5	0.3
9	−4.0	−4.0	0.0	−9.5	−9.8	0.3
10	−7.2	−7.2	0.0	−9.7	−10.1	0.3
Average			−0.01			0.28
	Lateral Field					
	Port‐Film	SimDRR	DRR‐SIM	Port‐Film	SimDRR	DRR‐SIM
BB#	*x* (cm)	*x* (cm)	*x* (cm)	*y* (cm)	*y* (cm)	*y* (cm)
1	6.1	6.1	0.0	6.4	6.3	0.1
2	3.7	3.8	−0.1	3.8	3.7	0.2
3	1.3	1.3	0.0	4.5	4.4	0.1
4	1.6	1.6	−0.1	0.9	0.8	0.2
5	−0.3	−0.2	−0.1	−2.6	−2.8	0.2
6	5.3	5.3	0.0	−3.0	−3.1	0.1
7	1.8	1.9	0.0	−6.1	−6.3	0.2
8	1.9	1.9	0.0	−7.4	−7.6	0.3
9	4.8	4.7	0.0	−7.7	−7.8	0.2
10	4.0	4.1	0.0	−9.6	−9.8	0.2
11	7.1	7.2	0.0	−8.7	−8.9	0.2
Average			−0.03			0.18

### 5. Monitoring individual markers

**Figure 5:**
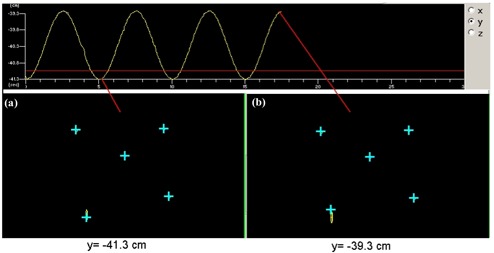
A snapshot of a computer screen for monitoring five IR reflective markers, four of which are static; one (inferior right) is moving in the cephalad‐caudad direction using a dynamic phantom. The blue crosses show the position of the markers at the time of a snapshot; the yellow oval shows the marker trajectories. The snapshots correspond to (a) the extreme inferior position of the IR marker and (b) the extreme superior position of the IR marker. The curve at the top shows the cephalad‐caudad trajectory of the IR marker.

Figure 5 shows a snapshot of a screen during monitoring the markers attached to a dynamic phantom. There is no motion observed for four markers attached to the body of the phantom, while a single inferior right marker is moving in the cephalad‐caudad direction. The motion curve displayed at the bottom of the screen indicates an amplitude of motion of 2 cm and a period of 5 s. This is in good agreement with the input parameters. This test illustrates the feasibility of using the current system for monitoring motion of an individual marker while relying on stable markers for setup verification.

### 6. Virtual portal vision performance

**Figure 6:**
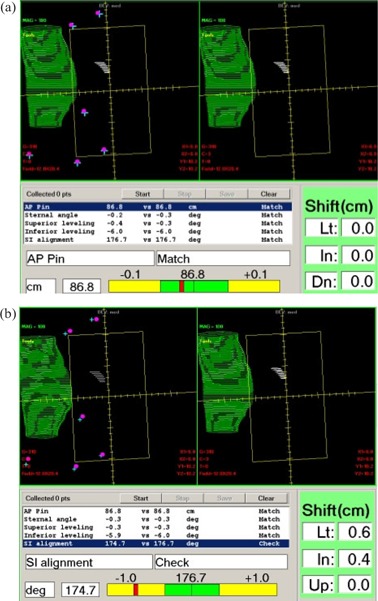
Virtual portal vision display for two different cases: (a) good setup and (b) incorrect setup. Two upper panels in each display show the BEV projections of the lung (green) and tumor cavity (white). The left panel displays the projections of the contours in the BEV using the real‐time positions of the markers determined by the IR system. The right panel is the reference BEV determined at the time of the simulation also with respect to the markers. The projections of the markers in BEV are shown as blue crosses (for real‐time marker location) and red circles (for marker locations at the time of the CT simulation). The lower‐left panel shows the patient setup parameters (clinical parameter indicator), and the lower‐right panel shows the table shift indicator. The clinical parameter indicator displays the list of the clinical parameters chosen to help with the setup for the specific site. For each parameter, two numbers are shown to display the value of the parameter in real time (first number on the line) and the value of the same parameter from the time of the CT simulation (second number on the line). For each parameter, the units are shown and an indicator whether the parameter is within the tolerance (matched) or needs to be checked. For a selected parameter, the information described above is highlighted at the bottom of the panel by a moving bar. The value calculated for this parameter at the CT simulation is shown in the center of the moving bar. The interval within the tolerance is shown in green, and the current value is shown as a sliding red bar.

Figures 6(a) and 6(b) illustrate the performance of the virtual portal vision application of the system's software. The two upper panels show BEV projections of the lung (green) and tumor cavity (white). The left panel displays the projections of the contours in the BEV using the real‐time positions of the markers determined by the IR system as described in the Appendix. The right panel is the reference BEV determined at the time of the simulation also with respect to the markers. The lower‐left panel shows the patient setup parameters, and the lower‐right panel shows the table shift indicator.

Figure 6(a) illustrates the case of a good setup. This is indicated by good agreement between the values for the clinical parameters at the treatment and CT simulation and by the table shift indicator showing that the table is within 1 mm from the ideal position in all directions. The good setup is also reflected in good agreement between real‐time and CT‐simulation BEV of contour projections (two upper panels). On both panels, the lung is 0.7 cm deep in the beam, and the tumor cavity is in a very similar position with respect to the beam central axis.

Figure 6(b) shows an example of a poor setup, which is indicated by dramatic differences in BEV projections of the contoured organs as well as large disagreement in clinical parameters with the “ideal” values. The difference in the BEV projections is well correlated with the direction of phantom misalignment. As indicated by the “Table shift” panel in Fig. 6(b), the table needs to be moved 0.6 cm to the left and 0.4 cm toward the gantry, which means that the phantom is actually shifted to the right and away from the gantry. For a left breast treatment, the shift of the patient to the right results in the lung moving away from the posterior field border, which is indeed the case in Fig. 6(b), and the shift of the phantom away from the gantry is well correlated with both the lung and the tumor cavity being shifted inferior with respect to the isocenter.

### 7. Respiratory control functions

**Figure 7:**
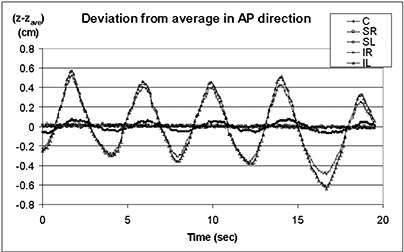
Deviation from average in the AP direction for five retroreflective markers monitored on a volunteer for 20 s. The locations of markers are as shown in Fig. 2(b): C, SR, SL, IR, and IL.

Figure 7 shows the AP displacement for the markers (C, SR, SL, IR, and IL) affixed to a person's skin and monitored by IR cameras for 20 s. As can be seen in the figure, the frequency of data collection and the precision of data recording are high enough to monitor respiration. What is also remarkable from the results in Fig. 7 is that the magnitude of the markers’ movements in the AP direction is greatly affected by the position of the marker on the chest: the markers in the lower chest area (IR and IL) have a much higher magnitude of movement with respiration, while more superiorly located markers (SR, SL, and C) are less affected by breathing. This result encourages us to focus on the system applications in the areas where some points can be used for the purpose of posture control, while other points can be used for respiratory control functions, similar to that reported in Refs. 7 to 9.

## IV. DISCUSSION

We have developed a system capable of interactively monitoring and controlling patient posture, position, and respiratory phase for radiation therapy treatment. This system consists of a set of two IR cameras, a set of retroreflective markers, and software to process the information obtained from the cameras and translate it into clinically relevant information. The marker coordinates obtained at the CT simulator are used as the “ideal” marker position. The location of the marker on the skin is either tattooed, marked with invisible ink, or a template of natural skin marks is made. These marks on the patient's skin are found before the treatment starts, and reflective markers are positioned on the marks. By comparing the marker position at the time of the treatment taken with an identical IR camera set installed in treatment room with its “ideal” position saved just before the CT scan, we obtain information necessary to adjust the patient's configuration. The setup verification can be done through a number of options. Among these options are evaluation of marker misposition in 2D planes, evaluation of port‐points in BEV, providing feedback in terms of clinical parameters, and by using a “virtual portal vision” display.

With a “virtual portal vision” application of our system, for CT simulation patients, we can instantaneously project a patient's organ contours in BEV (based on the position of the IR reference markers). By comparing these organ projections with DRRs from the CT simulator, we obtain instantaneous feedback on patient misalignment. The procedure is performed in real time and is almost instantaneous, therefore potentially reducing setup time.

### A. System performance

For a rigid phantom, this system has an overall intrinsic accuracy of ±1mm. When the phantom is moved from one room to another, the accuracy is better than ±2mm. We feel that this is acceptable for the purpose of guiding therapists to efficiently reproduce setups. We plan to implement this and test it systematically on a series of patients to evaluate the effectiveness of the system in the clinical setting. In the context of clinical applications, we further comment on the practical issues and capabilities of the system below.

### B. Practical implementation

The main objective of our system is to facilitate efficient and effective patient setup, that is, to improve current methods of patient positioning for radiation treatment while speeding up the procedure rather than slowing it down. To achieve this goal, we have developed Windows‐based software with a number of specific features: (1) a sequenced program control with a self‐guided menu to lead the user through the necessary steps; (2) a networked database system with all essential information being accessible from both CT and treatment rooms; (3) site‐specific templates with a predefined set of markers and a standard set of fields for a particular treatment site; and (4) posture control methods that translate raw marker coordinates into clinically relevant information. Below we discuss translation to clinical setup parameters in more detail.

When the markers are detected by the cameras, 3D coordinates (*x*,*y*,*z*) of the markers are obtained and can be used to verify the patient's posture and position. For clinical purposes, however, the coordinates of the points are difficult to use. Instead, these coordinates have to be translated into more clinically understandable parameters with which the therapists can effectively adjust the patient's setup. There are several advantages in using clinical parameters over raw coordinates of the markers in addition to being more understandable to clinical personnel. Since clinical parameters incorporate information of several markers’ positions, the errors generated during individual marker placement are diminished. Also, it is easier to identify the threshold values for these parameters based on previous clinical experience rather than for the marker coordinates.

The procedure to translate the marker coordinates into clinical parameters is site‐dependent and marker placement scheme‐dependent. Not all markers are used for calculations of each of the parameters. For example, for a breast cancer patient's setup, where markers are positioned as shown in Figs. 2(a) and 2(b), the critical parameters illustrated are sternum angle, leveling of the patient, and the focus‐to‐surface distance (FSD or PIN). The sternum angle is the slope of the chest‐wall in the superior‐inferior direction, and it is determined by two field points (superior corner and inferior corner). The leveling is the right‐to‐left rotation of the patient, and it is mainly determined by four posture points (SL, SR, IL, and IR). Finally, the PIN is calculated based on the center marker position.

In addition to providing feedback in terms of clinical parameters, a “virtual portal vision” display was developed to allow quick evaluation of patient setup in terms of organ projections in BEV. This was developed under the assumption of a strong correlation between internal anatomy and external marker location and was discussed in more detail in the Sections C and D.6 in Materials and Methods.

### C. Comparison to other systems

The differences between this new system and other IR‐technology systems used in radiation therapy are primarily in the application objectives for the system's design. In particular, the focus of our new system is on integration of position, posture, and respiratory control through IR‐system guidance, while other systems focus on either highly accurate position control,^(^ ^5^ ^,^ ^6^ ^,^ ^10^ ^–^ ^13^ ^)^ respiratory control alone, such as Varian's RPM,^(^ ^18^ ^–^ ^23^ ^)^ or elaborate setup verification,^(^ ^7^ ^–^ ^9^ ^)^ or on a combination of an IR system with either ultrasound^(^ ^14^ ^–^ ^16^ ^)^ or dual X‐ray tubes^(17)^ for a combination of posture and position control.

When the focus is put on precise isocenter correlation, such as for applications to stereotactic treatments,^(^ ^10^ ^–^ ^13^ ^)^ the rigid body model is applied and multiple markers are used to average the error of each individual marker localization and find the table adjustments based on isocenter position alone. In our system, we take a step farther away from assuming rigidity of the human body, and identify not only the isocenter position based on marker location, but also use multiple markers to verify the patient's body posture.

For respiratory control, it may be sufficient to monitor a single point on the patient's chest for respiratory phase identification; however, the setup uncertainties between one breathing phase and another become a problem. By combining respiratory control function with the setup control, we develop a system that may be potentially more accurate and reliable for gating or breath‐hold applications.

A system similar to the current one was developed by Baroni et al.^(^ ^7^ ^–^ ^9^ ^)^ for a detailed setup verification. This system was used to analyze the setup errors on a number of breast cancer patients, and they reported dosimetric consequences of these errors. In our new system, we recognize the importance of a more comprehensive setup verification and focus on development of specific features of the system that can integrate the setup with respiratory and position control and simplify the setup procedure. One of the unique features of our system is a “virtual portal vision” application that can potentially decrease the setup time and limit X‐ray port‐film if proved efficient.

This new system is a variation of the sonic system that was used at our institution for localizing points to be tattooed on the patients.^(24)^ While the original system^(24)^ was designed to use sonic localizers in conjunction with a digitizing tool for a single point localization, our new system focuses on multiple point recognition and use of these points for setup control.

## V. CONCLUSIONS

The present optical system based on IR retroreflective technology is a promising tool to interactively monitor and control the patient's posture and position for radiation therapy treatment. This system consists of an IR camera set, several retroreflective markers, and a PC with designated software to process information obtained from the cameras. The speed of data acquisition and processing is high enough to allow monitoring of the patient in real time and to obtain adequate information about the patient's movement and breathing. With a built‐in system of simple instructions and voice commands, together with voice‐activated operation, the system facilitates interactive patient posture adjustment. The accuracy of the system is well within the acceptable uncertainty for typical radiation therapy applications. We intend to evaluate the system in a clinical setting for initial setup as well as for monitoring patients during treatment.

## ACKNOWLEDGMENT

We are thankful to Dr. A.M. Allen and Dr. G.M. Makrigiorgos for critical comments and suggestions.

## Coordinate transformation for treatment room/CT simulator organ contour displacement

As was noted before, we describe patient configuration in the room in terms of position (table shift) and posture (marker set orientation relative to central marker). The same is true for individual organ position description. The table shift is calculated based on coordinates of the central marker. To calculate coordinate transformations for the rotational displacement of the marker set in the treatment room with respect to that in the CT simulator, the coordinate systems are defined for both the treatment room and the CT simulator. Below, the superscript RT corresponds to the coordinate system of the treatment room, and the symbol CT corresponds to the coordinate system of the CT simulator. Coordinate systems are chosen to be identical to our planning system, that is, the *x*‐axis runs from the left of the patient to the right, the *y*‐axis runs from inferior to superior of the patient, and the *z*‐axis runs in the posterior‐anterior direction, provided that the patient is in the supine position, head first. The centers of both coordinate systems are defined to be at the isocenter of the gantry for both rooms.

Special care is taken to ensure that the position of the central marker (marker C in Fig. 2(b), to which PIN is measured) is exactly the same in the treatment room as at the CT simulation. We therefore assume that this point is defined with the highest precision and calculate rotation of the marker set around the marker C. For each marker *k* on the patient, we can define the vector for the marker position in the treatment room $R_{k}^{\text{RT}}$ with respect to the marker C, and the vector for the marker *k* position in the simulator $R_{k}^{\text{CT}}$ with respect to the marker C. Rotational angles around the marker C are as follows: $\phi_{k}^{i}$ (around the *x*‐axis), $\varphi_{k}^{i}$ (around the *y*‐axis), and $\psi_{k}^{i}$ (around the *z*‐axis), where i=RT or CT. They are calculated as

$$\begin{array}{rl} & \phi_{k}^{i}=\text{arctan}(z_{k}^{i}/y_{k}^{i})\\ & \varphi_{k}^{i}=\text{arctan}(x_{k}^{i}/z_{k}^{i})\\ & \psi_{k}^{i}=\text{arctan}(y_{k}^{i}/x_{k}^{i}) \end{array}$$

Rotational misplacement of the marker set in the treatment room (compared to CT simulation) is calculated by averaging differences in rotational angles for individual markers:

$$\begin{array}{rl} & \Phi =\frac{1}{\left(n-1\right)}\sum_{k=2}^{n}(\phi_{k}^{\text{RT}}-\phi_{k}^{\text{CT}}),\\ & \Theta =\frac{1}{\left(n-1\right)}\sum_{k=2}^{n}(\varphi_{k}^{\text{RT}}-\varphi_{k}^{\text{CT}}),\\ & \Psi =\frac{1}{\left(n-1\right)}\sum_{k=2}^{n}(\psi_{k}^{\text{RT}}-\psi_{k}^{\text{CT}}), \end{array}$$

where *n* is the number of markers affixed to the patient. Note that we do not include marker C in the sum for calculating the angles, since all angles are defined with respect to this central marker. Rotation matrixes are then defined as follows:

$$\begin{array}{rl} & R_{x}=\left(\begin{array}{ccc} 1 & 0 & 0\\ 0 & \cos\Phi & \sin\Phi \\ 0 & -\sin\Phi & \cos\Phi \end{array}\right),\\ & R_{y}=\left(\begin{array}{ccc} \cos\Theta & 0 & -\sin\Theta \\ 0 & 1 & 0\\ \sin\Theta & 0 & \cos\Theta \end{array}\right),\\ & R_{z}=\left(\begin{array}{ccc} \cos\Psi & \sin\Psi & 0\\ -\sin\Psi & \cos\Psi & 0\\ 0 & 0 & 1 \end{array}\right). \end{array}$$

These rotations are applied to the organ (lung and heart) contours obtained from CT simulation, $C^{\text{CT}}$, to display these contours at treatment time, $C^{\text{RT}}$,

$$C^{\text{RT}}=R_{x}R_{y}R_{z}C^{\text{CT}}.$$

After the initial coordinate transformation is performed, an additional transformation, $R_{\text{BEV}}$, is applied to display the organ contours in the beam's‐eye‐view:

$$C^{\text{BEV}}=R_{\text{BEV}}C^{\text{RT}}.$$

The transformation $R_{\text{BEV}}$ is described elsewhere.^(24)^

In application to breast treatments, the procedure described above immediately allows one to compare the lung position at treatment time relative to the field edge and make decisions about possible adjustments to patient posture. The main advantage of this procedure is that it is performed in real time and can save extra portal‐films in the case of noticeable patient misalignment. The drawback is that we assume that the correlation between lung shape and skin surface remains constant.

## References

[1] Verhey LJ . Immobilizing and positioning patients for radiotherapy. Semin Radiat Oncol. 1995;5:100–114. 10717133 10.1054/SRAO00500100

[2] Hurkmans CW , Remeijer P , Lebesque JV , Mijnheer BJ . Setup verification using portal imaging: Review of current clinical practice. Radiother Oncol. 2001;58:105–120. 11166861 10.1016/s0167-8140(00)00260-7

[3] Langen KM , Jones DTL . Organ motion and its management. Int J Radiat Oncol Biol Phys. 2001;50:265–278. 11316572 10.1016/s0360-3016(01)01453-5

[4] Meeks SL , Tome WA , Willoghby TR , et al. Optically guided patient positioning techniques. Semin Radiat Oncol. 2005;15:192–201. 15983944 10.1016/j.semradonc.2005.01.004

[5] Soete G , Van de Steene J , Verellen D , et al. Initial clinical experience with infrared‐reflecting skin markers in the positioning of patients treated by conformal radiotherapy for prostate cancer. Int J Radiat Oncol Biol Phys. 2002;52:694–698. 11849791 10.1016/s0360-3016(01)02642-6

[6] Soete G , Verellen D , Michielsen D , et al. Clinical use of stereoscopic X‐ray positioning of patients treated with conformal radiotherapy for prostate cancer. Int J Radiat Oncol Biol Phys. 2002;54:948–952. 12377349 10.1016/s0360-3016(02)03027-4

[7] Baroni G , Ferrigno G , Pedotti A . Implementation and application of real‐time motion analysis based on passive markers. Med Biol Engineer Comput. 1998;36:693–703. 10.1007/BF02518871 10367459

[8] Baroni G , Ferrigno G , Orecchia R , Pedotti A . Real‐time three dimensional motion analysis for patient positioning verification. Radiother Oncol. 2000;54:21–27. 10719696 10.1016/s0167-8140(99)00166-8

[9] Baroni G , Ferrigno G , Orecchia R , Pedotti A . Real‐time opto‐electronic verification of patient position in breast cancer radiotherapy. Comput Aided Surg. 2000;5:296–306. 11029162 10.1002/1097-0150(2000)5:4<296::AID-IGS8>3.0.CO;2-I

[10] Bova FJ , Buatti JM , Friedman WA . The University of Florida frameless high‐precision stereotactic radiotherapy system. Int J Radiat Oncol Biol Phys. 1997;38:875–882. 9240657 10.1016/s0360-3016(97)00055-2

[11] Tome WA , Meeks SL , Buatti JM . A high‐precision system for conformal intracranial radiotherapy. Int J Radiat Oncol Biol Phys. 2000;47:1137–1143. 10863087 10.1016/s0360-3016(00)00502-2

[12] Ryken TC , Meeks SL , Pennington EC . Initial experience with frameless stereotactic radiosurgery. Int J Radiat Oncol Biol Phys. 2001;51:1152–1158. 11704340 10.1016/s0360-3016(01)01756-4

[13] Meeks SL , Buatti JM , Bouchet LG , et al. Ultrasound‐guided extracranial radiosurgery: Technique and application. Int J Radiat Oncol Biol Phys. 2003; 55:1092–1101. 12605989 10.1016/s0360-3016(02)04406-1

[14] Bouchet LG , Meeks SL , Bova FJ . 3D ultrasound image guidance for high precision extracranial radiosurgery and radiotherapy. Radiosurgery 2002;4:262–278.

[15] Wang LT , Solberg TD , Medin PM , Boone R . Infrared patient positioning for stereotactic radiosurgery of extracranial tumors. Comput Biol Med. 2001;31:101–111. 11165218 10.1016/s0010-4825(00)00026-3

[16] Tome WA , Meeks SL , Orton NP , Bouchet LG , Bova FJ . Commissioning and quality assurance of an optically guided three‐dimensional ultrasound target localization system for radiotherapy. Med Phys. 2002;29:1781–1788. 12201425 10.1118/1.1494835

[17] Verellen D , Soete G , Linthout N . Quality assurance of a system for improved target localization and patient setup that combines real‐time infrared tracking and stereoscopic X‐ray imaging. Radiother Oncol. 2003;67:129–141. 12758249 10.1016/s0167-8140(02)00385-7

[18] Kubo HD , Hill BC . Respiration gated radiotherapy treatment: A technical study. Phys Med Biol. 1996;41:83–91. 8685260 10.1088/0031-9155/41/1/007

[19] Ford EC , Mageras GS , Yorke E , et al. Evaluation of respiratory movement during gated radiotherapy using film and electronic portal imaging. Int J Radiat Oncol Biol Phys. 2002;52:522–531. 11872300 10.1016/s0360-3016(01)02681-5

[20] Mageras GS , Yorke E , Rozenzweig K , et al. Fluoroscopic evaluation of diaphragmatic motion reduction with a respiratory gated radiotherapy system. J Appl Clin Med Phys. 2001;2:191–200. 11686740 10.1120/jacmp.v2i4.2596 PMC5726007

[21] Ramsey CR , Scaperoth D , Arwood D . Clinical experience with a commercial respiratory gating system. Int J Radiat Oncol Biol Phys. 2000;48:164–165.

[22] Berson AM , Emery R , Ng T , Rodriguez L , Sanghavi S . Clinical experience using respiratory gated radiation therapy for tumors in the chest, upper abdomen, and breast. Int J Radiat Oncol Biol Phys. 2002;54:195–196. 15380575 10.1016/j.ijrobp.2004.03.037

[23] Jiang S , Neicu T , Chen GT . Gated motion adaptive therapy (GMAT): A new modality for treating mobile tumors. Int J Radiat Oncol Biol Phys. 2002;54:195.12182992

[24] Lu HM , Chin L . Virtual light field projection for CT‐simulation. Med Phys. 1999;26:1222–1229. 10435521 10.1118/1.598616

[25] Wiles AD , Thompson DG , Frantz DD . Accuracy assessment and interpretation for optical tracking systems. To be published in Medical Imaging; from www.ndigital.com.

